# Sporadic Fundic Gland Polyposis Associated With Long-Term Proton Pump Inhibitor Use: A Case Report

**DOI:** 10.7759/cureus.88557

**Published:** 2025-07-22

**Authors:** Huanghuan Li, Tze Tong Tey

**Affiliations:** 1 Gastroenterology, Sengkang General Hospital, Singapore, SGP

**Keywords:** fundic gland polyp, fundic gland polyposis syndrome, gastric polyps, proton pump inhibitors, upper endoscopy

## Abstract

Fundic gland polyps (FGPs) are the most common type of gastric polyps and are frequently discovered incidentally during upper endoscopy. Long-term use of proton pump inhibitors (PPIs) has been associated with an increased risk of developing FGPs. We present a case of PPI-induced FGP in a 78-year-old woman who had been taking omeprazole daily for 15 years and was referred for evaluation of anemia. Gastroscopy revealed innumerable FGPs in the gastric body. Histological analysis demonstrated dilated glands within the fundic mucosa, consistent with FGPs. These polyps are typically small and benign and located in the gastric corpus and fundus. While FGPs are generally non-neoplastic, discontinuation of PPI therapy should be considered on a case-by-case basis. Indications for long-term PPI use should be regularly assessed. Unnecessary PPI therapy should be discontinued when appropriate.

## Introduction

Gastric polyps are frequently discovered incidentally during upper gastrointestinal endoscopy. Among them, fundic gland polyps (FGPs) are the most common, accounting for approximately 47% of all gastric polyps [1]. First described in 1977, FGPs are typically small, ranging from 1 to 5 mm in diameter, and are predominantly located in the gastric body and fundus [2]. Endoscopically, they present as sessile lesions with a smooth, shiny, and translucent surface [3]. These polyps are usually hemispherical and either paler than or similar in color to the surrounding mucosa. Their surface often exhibits tiny surface blood vessels [4].

Histologically, FGPs consist of cystically dilated oxyntic glands lined by attenuated chief, parietal, and mucous neck cells, and their surface is covered by gastric foveolar epithelium [5]. Most patients with FGPs are asymptomatic. The risk of malignant transformation is very low, estimated to be less than 1% in polyps larger than 1 cm; hence, routine surveillance is generally not recommended [6].

The American Society for Gastrointestinal Endoscopy (ASGE) guidelines published in 2015 recommend resection of FGPs ≥1 cm due to the slightly increased risk of dysplasia associated with larger lesions [7]. While FGPs can be solitary, the presence of multiple polyps should prompt evaluation for an underlying hereditary polyposis syndrome. These include familial adenomatous polyposis (FAP), MUTYH-associated polyposis, and gastric adenocarcinoma and proximal polyposis of the stomach (GAPPS). In these syndromic cases, the risk of dysplasia is significantly higher, ranging from 25% to 62%, with potential for progression to gastric adenocarcinoma [8].

Syndromic FGPs typically occur in younger individuals, often under 40 years of age, and may be associated with duodenal adenomas [6]. Although these hereditary polyposis syndromes are rare, there has been a notable increase in the prevalence of sporadic FGPs in recent years, largely attributed to extensive long-term proton pump inhibitor (PPI) use [9].

This case report aims to present a rare case of fundic gland polyposis likely attributed to long-term PPI use, in the absence of a hereditary polyposis syndrome. Through this case, we hope to highlight the importance of critical assessment of PPI use in patients and for withdrawal of PPI if it is not required.

## Case presentation

A 78-year-old woman with a medical history of hypertension, hyperlipidemia, and prior ischemic stroke was referred for evaluation of anemia. She was asymptomatic at presentation and specifically denied gastrointestinal symptoms or a family history of gastrointestinal malignancy. Her long-term medications included omeprazole 20 mg daily, which she had been taking for 15 years for gastrointestinal prophylaxis for aspirin use.

**Figure 1:**
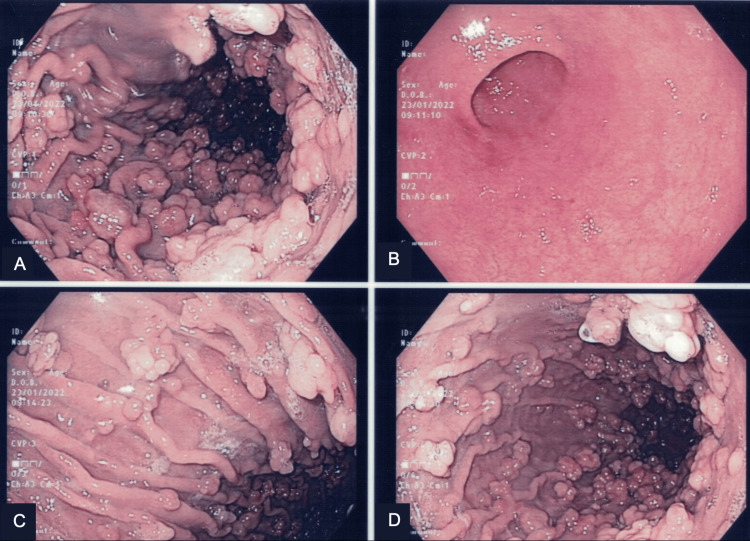
Gastroscopy finding. A, C, D: Innumerable fundic gland polyps carpeting gastric body. B: The antrum was normal.

On examination, there were no significant findings. Laboratory evaluation revealed a hemoglobin level of 11.2 g/dL. Esophagogastroduodenoscopy (EGD) demonstrated innumerable sessile sub-centimeter polyps carpeting the entire gastric body, with sparing of the antrum (Figure 1). The surrounding mucosa appeared normal, without evidence of atrophy, and no duodenal polyps were observed. The endoscopic appearance was characteristic of FGPs.

**Figure 2:**
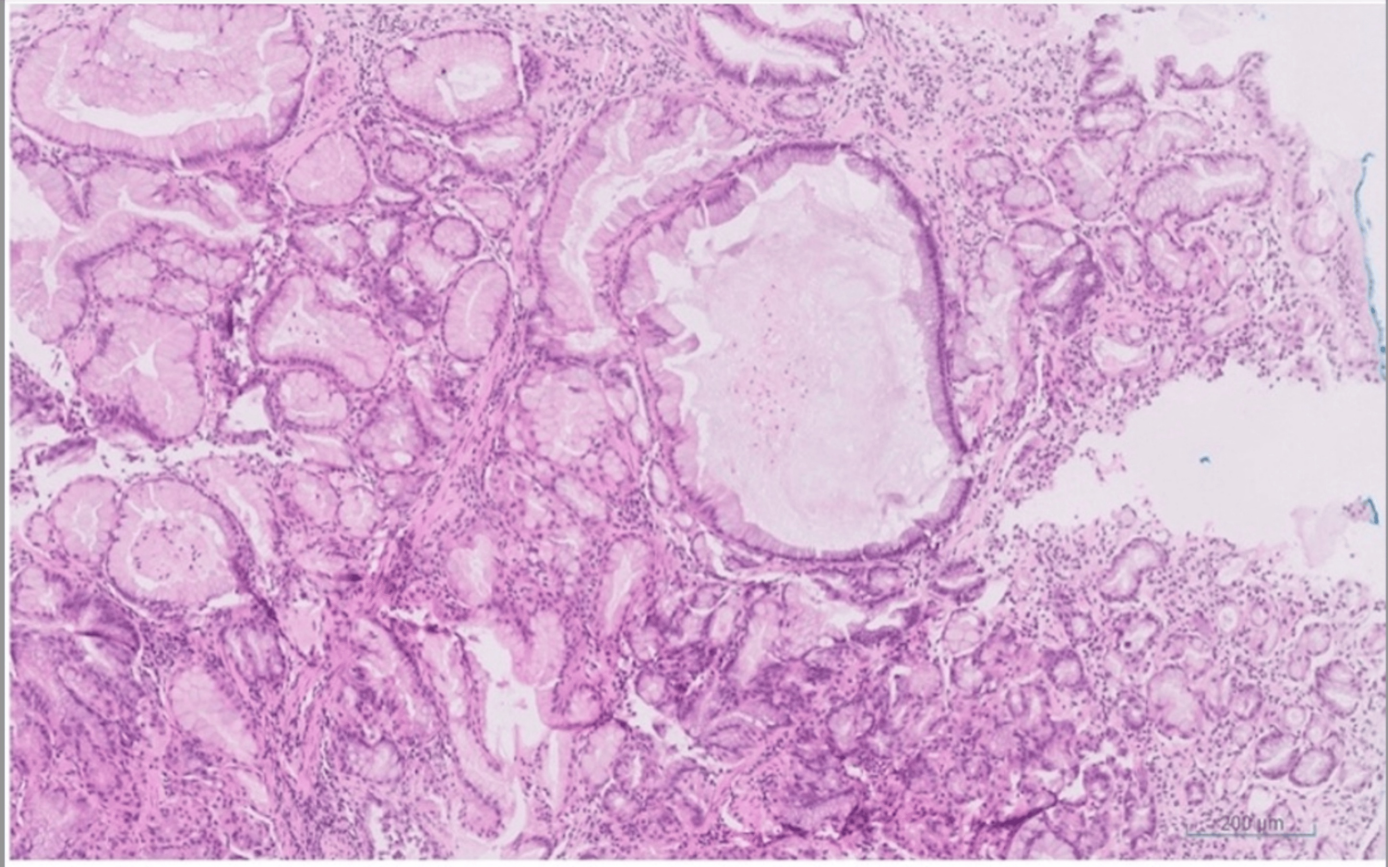
Histological finding. Fundic gland mucosa with dilated glands. No dysplasia (hematoxylin-eosin stain).

**Figure 3:**
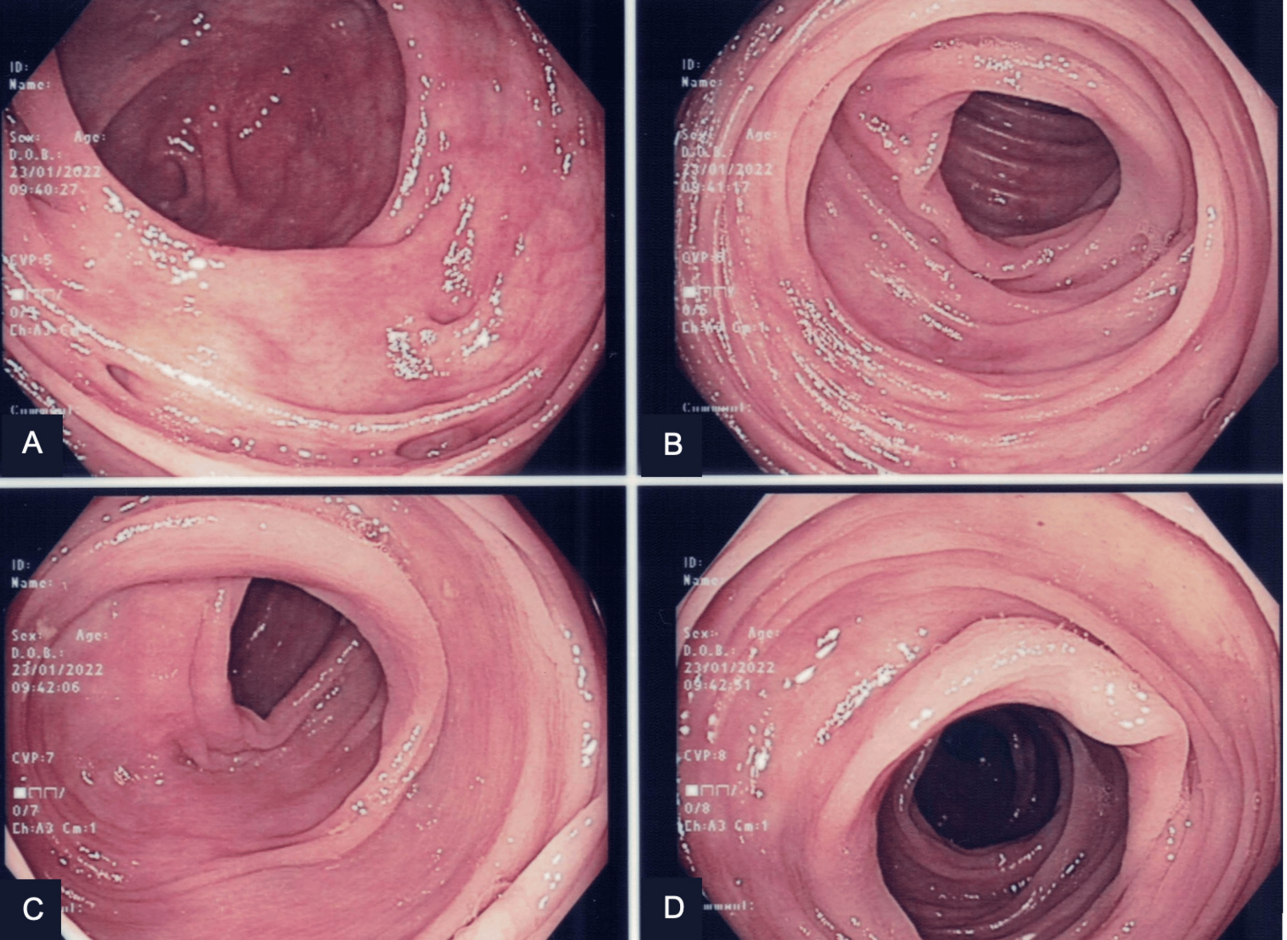
Colonoscopy findings. A: Multiple diverticula were observed in the ascending colon. B,C,D: No colonic polyps were detected throughout the entire colon.

Histopathological examination of the biggest polyps, 1 cm in size, revealed specialized fundic mucosa with cystically dilated glands, consistent with FGPs. There was no evidence of dysplasia, mucosal inflammation, or *Helicobacter pylori* infection (Figure 2). Colonoscopy was subsequently performed and revealed no colonic polyps (Figure 3).

Based on the endoscopic, histologic, and clinical findings, the fundic gland polyposis was attributed to prolonged PPI use.

Following discussion, the patient opted to continue the PPI therapy for gastric prophylaxis in the context of ongoing aspirin use, which remains indicated due to a prior history of stroke. Anaemia has remained stable on follow-up. 

## Discussion

The majority of gastric polyps are benign with a low risk of malignant transformation. Gastric adenomatous polyps of any size and hyperplastic polyps greater than 5 mm in size are particularly of higher concern and should be resected whenever possible. Patients with these polyps are also at increased risk for gastric premalignant conditions (GPMCs), such as gastric atrophy, intestinal metaplasia, and dysplasia, which necessitate careful mucosal assessment. This approach has been emphasized in the recent updates to the quality indicators for upper gastrointestinal endoscopy published in the American Journal of Gastroenterology [10].

By contrast, FGPs carry a negligible risk of malignant progression, and extensive resection is not typically required. This highlights the importance of accurate endoscopic characterization of gastric polyps during initial upper endoscopy.

There has been a notable rise in the prevalence of sporadic FGPs, primarily associated with prolonged PPI use [9]. The proposed mechanism involves hypergastrinemia secondary to acid suppression, which in turn promotes enterochromaffin-like (ECL) cell proliferation and oxyntic gland hyperplasia. A prospective study by Jalving et al. involving 599 patients undergoing upper endoscopy demonstrated that prolonged PPI use significantly increased the risk of developing FGPs. Patients on PPIs for one to five years had an odds ratio (OR) of 2.2 (95% CI: 1.3-3.8), while those on PPIs for over five years had an OR of 3.8 (95% CI: 2.2-6.7) [11].

Sporadic FGPs are considered benign and are often associated with activating mutations in the β-catenin gene [3]. These polyps rarely show dysplasia, and their identification should prompt a review of the patient’s medication history, particularly long-term PPI use. Elevated serum gastrin in this context is believed to function as a trophic factor for oxyntic mucosa, further contributing to polyp development.

Although the malignant potential of FGPs is low, they can, albeit rarely, be associated with upper gastrointestinal bleeding. In a retrospective study of 5,000 patients, gastric polyps were identified as the source of bleeding in 0.28% of cases [12]. Tanaka et al. also reported a case of PPI-induced FGPs complicated by hematemesis, with polyp regression observed following discontinuation of the PPI [13]. Hence, the indication for long-term PPI therapy should be regularly reviewed, and unnecessary use should be discontinued. 

## Conclusions

FGPs are a common type of gastric polyp, often found incidentally. Their association with long-term PPI use is increasingly recognized. In rarer cases, fundic gland polyposis can also be associated with long-term PPI use after ruling out familial polyposis syndrome. In our case, the patient remained asymptomatic with no dysplasia or malignant changes observed on histology. Given the benign nature of the polyps, no endoscopic resection was required. Clinicians should remain vigilant about reviewing the indications for long-term PPI use and stop PPI when required.

## References

[1] Stolte M, Sticht T, Eidt S, Ebert D, Finkenzeller G (1994). Frequency, location, and age and sex distribution of various types of gastric polyp. Endoscopy.

[2] Freeman HJ (2008). Proton pump inhibitors and an emerging epidemic of gastric fundic gland polyposis. World J Gastroenterol.

[3] Islam RS, Patel NC, Lam-Himlin D, Nguyen CC (2013). Gastric polyps: a review of clinical, endoscopic, and histopathologic features and management decisions. Gastroenterol Hepatol (N Y).

[4] Genta RM, Schuler CM, Robiou CI, Lash RH (2009). No association between gastric fundic gland polyps and gastrointestinal neoplasia in a study of over 100,000 patients. Clin Gastroenterol Hepatol.

[5] Levy MD, Bhattacharya B (2015). Sporadic fundic gland polyps with low-grade dysplasia: a large case series evaluating pathologic and immunohistochemical findings and clinical behavior. Am J Clin Pathol.

[6] Adam B, Pech O, Steckstor M, Tannapfel A, Riphaus A (2013). Sporadic fundic gland polyps. Video J Encyclopedia GI Endoscopy.

[7] Evans JA, Chandrasekhara V, Chathadi KV (2015). The role of endoscopy in the management of premalignant and malignant conditions of the stomach. Gastrointest Endosc.

[8] Wu TT, Kornacki S, Rashid A, Yardley JH, Hamilton SR (1998). Dysplasia and dysregulation of proliferation in foveolar and surface epithelia of fundic gland polyps from patients with familial adenomatous polyposis. Am J Surg Pathol.

[9] Marcial MA, Villafaña M, Hernandez-Denton J, Colon-Pagan JR (1993). Fundic gland polyps: prevalence and clinicopathologic features. Am J Gastroenterol.

[10] Yadlapati R, Early D, Iyer PG, Morgan DR, Sengupta N, Sharma P, Shaheen NJ (2025). Quality indicators for upper GI endoscopy. Am J Gastroenterol.

[11] Jalving M, Koornstra JJ, Wesseling J, Boezen HM, DE Jong S, Kleibeuker JH (2006). Increased risk of fundic gland polyps during long-term proton pump inhibitor therapy. Aliment Pharmacol Ther.

[12] Saowaros V, Udayachalerm W, Wee-Sakul B (1994). Causes of upper gastrointestinal bleeding in Thai patients: review of 5,000 upper gastrointestinal endoscopy. J Med Assoc Thai.

[13] Tanaka M, Kataoka H, Yagi T (2019). Proton-pump inhibitor-induced fundic gland polyps with hematemesis. Clin J Gastroenterol.

